# An Examination of Clinician Responses to Problem Gambling in Community Mental Health Services

**DOI:** 10.3390/jcm9072075

**Published:** 2020-07-01

**Authors:** Victoria Manning, Nicki A. Dowling, Simone N. Rodda, Ali Cheetham, Dan I. Lubman

**Affiliations:** 1 Turning Point, Eastern Health, 110 Church Street, Richmond, VIC 3121, Australia; s.rodda@auckland.ac.nz (S.N.R.); alisonc@turningpoint.org.au (A.C.); dan.lubman@monash.edu (D.I.L.); 2 Monash Addiction Research Centre and Eastern Health Clinical School, Monash University, 5 Arnold St, Box Hill, VIC 3128, Australia; 3 School of Psychology, Deakin University, 1 Gheringhap St, Geelong VIC 3220, Australia; nicki.dowling@deakin.edu.au; 4 Melbourne Graduate School of Education, University of Melbourne, Kwong Lee Dow Building, 234 Queensberry Street, Parkville, VIC 3053, Australia; 5 School of Population Health, Faculty of Medical and Health Sciences, 216 Morrin Road, Auckland 1142, New Zealand

**Keywords:** problem gambling, screening and assessment, comorbidity, clinician attitudes, clinical practice

## Abstract

Gambling problems commonly co-occur with other mental health problems. However, screening for problem gambling (PG) rarely takes place within mental health treatment settings. The aim of the current study was to examine the way in which mental health clinicians respond to PG issues. Participants (*n* = 281) were recruited from a range of mental health services in Victoria, Australia. The majority of clinicians reported that at least some of their caseload was affected by gambling problems. Clinicians displayed moderate levels of knowledge about the reciprocal impact of gambling problems and mental health but had limited knowledge of screening tools to detect PG. Whilst 77% reported that they screened for PG, only 16% did so “often” or “always” and few expressed confidence in their ability to treat PG. However, only 12.5% reported receiving previous training in PG, and those that had, reported higher levels of knowledge about gambling in the context of mental illness, more positive attitudes about responding to gambling issues, and more confidence in detecting/screening for PG. In conclusion, the findings highlight the need to upskill mental health clinicians so they can better identify and manage PG and point towards opportunities for enhanced integrated working with gambling services.

## 1. Introduction

Gambling disorder (formerly pathological gambling) has been classified as an addiction and related disorder in the fifth edition of the Diagnostic and Statistical Manual of Mental Disorders [1]. Many jurisdictions, including Australia, that have adopted a public health framework that conceptualizes gambling problems across a continuum of risk employ the term “problem gambling” (PG) to refer to all forms of gambling that lead to adverse consequences for the gambler, others, or the community [2]. Globally, it has been estimated that between 0.12% and 5.8% of adults experience past-year problems due to gambling across all countries [3]. Within Australia, approximately 0.4–0.6% of the adult population experience gambling problems each year, with an additional 1.9–3.7% reporting moderate-risk gambling and 3.0–7.7% low-risk gambling [4,5]. Moreover on a per capita basis, Australians spend more money on gambling than any other country; it has been estimated that the average Australian adult lost $1292 to gambling in 2017–2018 [6]. The most recent population prevalence survey of gambling in Victoria, the Australian state in which the current study was conducted, revealed that 0.7% of the population reported problem gambling, 2.4% reported moderate-risk gambling, and 6.7% reported low-risk gambling. [7]

Systematic reviews and meta-analyses suggest that comorbid mental health disorders are elevated in people with problem gambling in the community, with the highest mean prevalence for nicotine dependence (60.1%), followed by substance use disorder (57.5%), mood disorder (37.9%), and anxiety disorder (37.4%) [8]. Although it has been argued that potential sample selection bias in some prevalence surveys may underestimate the prevalence of gambling disorders and overestimate psychiatric comorbidities [9], high rates of comorbid substance use, mood and anxiety disorders, and personality disorders have also been consistently reported among those seeking treatment for PG [10]. A range of psychiatric disorders are also positively associated with the subsequent development of PG [11]. For example, a recent longitudinal investigation into the reciprocal association between PG and mental health in the Australian community provided evidence that depression and generalized anxiety contribute to the progression and development of gambling problems [12]. Moreover, there is mounting international evidence that PG commonly co-occurs in people seeking treatment for affective disorders [13], substance use disorders [14], psychotic disorders [15,16], post-traumatic stress disorder (PTSD) [17], and bipolar disorder [18]. Indeed, in a sample of 837 patients with various mental health disorders in Victoria, Australia, we found that the prevalence of PG (measured using the most widely used screening tool, the Problem Gambling Severity Index (PGSI)) [19] was around 8 times higher than in the general population at 6.3% [20].

The quantification of harm caused by PG suggests that its impact on quality of life is comparable to the impact of moderate to severe alcohol use disorder and major depressive disorder [12]. Despite this, most problem gamblers are unwilling to seek help and only a minority receive professional treatment [12]. Consequentially, healthcare professionals in mental health settings have a key role in the early identification and management of PG. Responding to PG in mental health patients is particularly pertinent given that this group frequently experiences marginalization and disadvantage, including high rates of stigma, isolation, and unemployment, low income or reliance on financial support from government, and reduced social support [21,22]. Mental health problems may also be underpinned by shared vulnerabilities that increase the risk of PG, such as high impulsivity, increased sensitivity to reward, emotional dysregulation, and cognitive impairment [23,24,25]. For individuals with comorbid mental health problems, these characteristics may drive extended periods of play and make it difficult to keep track of time and amount of money spent when gambling, leading to losses and financial stress [26,27].

Whilst routine screening of mental health service users could facilitate the early identification and treatment of PG [28], international research indicates that screening for PG rarely takes place in treatment settings [29,30,31] more broadly. As such, gambling problems often remain undetected and untreated until associated problems (e.g., financial and relationship difficulties, etc.) become more severe and pronounced [32]. To date, the limited research in this area has focused on general practitioners (GPs), with one study reporting that whilst GPs were aware of the existence and potential impact of PG on their patients, PG screening was not systematic and knowledge of adequate treatments or referral methods was poor [33]. Researchers elsewhere have also recognized the crucial role GPs play in the detection of gambling problems (e.g., in the UK, Canada, and Australia [29,31,34]) as well as some of the challenges and barriers [35]. However, there has been limited research exploring barriers to screening among clinicians working in mental health treatment settings who are likely to encounter a significant number of patients with PG. In a qualitative study exploring the barriers and facilitators to responding to PG among 30 mental health clinicians working across 11 services in Victoria, Australia, we found that competing clinical priorities (more pertinent/immediate risks), lack of access to appropriate screening tools and resources, and training deficits emerged as key themes [36]. However, the manner in which mental health clinicians currently respond to PG among their patients remains unknown. Information on how regularly and confidently clinicians identify PG, refer for specialist treatment, or manage PG as part of their own care plan is pivotal to the planning of effective interagency working relationships and treatment approaches that ensure optimal and timely delivery of care to this vulnerable population.

The objectives of this study were to determine current practices among mental health clinicians across a range of Victorian public and private adult mental health services. The study also sought to examine clinicians’ attitudes towards addressing gambling problems among patients, the estimated prevalence of PG among their caseloads, perceived need for routine screening, and the need for training in responding to gambling issues.

## 2. Experimental Section

### 2.1. Design and Sample

Participants (*n* = 281) were recruited from a range of adult mental health services in Victoria, Australia, as part of a larger study on PG in people seeking treatment for mental illness [37]. These services are focused on delivering treatment for a broad range of mental health disorders and are distinct from Gambling Help and addiction services in Victoria that specifically provide clinical care for individuals with gambling and substance use disorders. Sites were selected as representative of the wide range of services available in Australia, including state-funded (public) community mental health services (inner metropolitan, outer metropolitan, and regional sites), outpatient private mental health clinics (where patient fees are mostly covered through Medicare, Australia’s universal healthcare scheme), a large statewide Psychiatric Disability Rehabilitation and Support Service (PDRSS), and primary care sites. Participants included clinicians without specific gambling expertise who work at the forefront of clinical services and respond to a large number of adult mental health patients (e.g., nurses, psychologists, case managers, and doctors). Recruitment took place between September 2014 and March 2015. In total, 70% of eligible clinicians were sampled.

### 2.2. Measures

To explore the knowledge, attitudes, and experience of mental health practitioners across the targeted services, a self-completion questionnaire was developed. Following a section on demographic characteristics, participants were assessed for knowledge of gambling and mental health (3 items), screening and assessment (7 items), and attitudes towards gambling and mental illness and role congruence and methods for exploring gambling behaviors, including the use of screening tools (12 items). Clinicians were then asked what they do upon identifying a patient with a gambling problem (single item, multiple-choice options) and were provided with a list of mental health conditions in order to indicate which conditions they believe most commonly co-occur with gambling problems. This was followed by a section on referral practices (7 items) and a final section on treatment (6 items). Questions were presented through a combination of Likert scales, dichotomous/multiple response options, established/standardized measures, and targeted open-ended questions. This included some adapted items from the 19-item scale used to explore the feasibility and acceptability of a mental health screening tool among healthcare workers in the alcohol and drug sector [38]. Following piloting of the three-page questionnaire and amendments, a paper-and-pencil version was finalized and an online version was developed using Qualtrics. The survey was anonymous and took approximately 15 min to complete.

### 2.3. Recruitment/Procedure

Researchers worked with the service/practice managers to identify the best approaches to promote the survey. Surveys were completed either in team meetings or online during clinicians’ own time. The research team undertook a series of briefings (e.g., at departmental meetings) at each service to introduce the project and explain how the findings could benefit the service. In some services, hard copies of the questionnaire were administered and completed during a staff meeting and returned directly to the researchers or collected at a later date. In other services, an email invitation, along with the weblink to the online survey, was sent to all eligible clinicians via the practice manager on behalf of the research team to invite eligible staff to participate. Researchers worked closely with the team leaders to monitor survey completion rates and send reminder emails, with follow-up phone calls or visits undertaken to increase the response rate. As an incentive, staff had the opportunity to enter into a competition to win one of three tablets upon completion of the survey. Over 70% of eligible clinicians were sampled. The study was given approval by the Eastern Health Human Research Ethics Committee (reference number LR120/1314), and additional ethical review was undertaken and approval granted by sites not directly covered by the Eastern Health Human Research Ethics Committee.

### 2.4. Data Analysis

Statistical analyses with pooled data from the eight mental health settings (from 13 different sites) were conducted using SPSS 22.0 (IBM Corp, Armonk, NY, USA). Descriptive statistics were computed for participant characteristics (demographics) and practices (e.g., screening, referral, and treatment) reported as percentages to indicate the proportion of services routinely screening, referring, and treating patients with gambling problems. Associations between gambling practices represented by categorical data (e.g., screening/referral confidence or frequency response options) and past training in gambling were explored using Pearson’s chi-square tests. Where appropriate, adjacent categories were collapsed into smaller categories when sample size per cell was too small to fulfill the necessary Pearson’s chi-square requirements. For example, 5-point Likert scale items (e.g., statements assessing knowledge about gambling problems and mental illness) were collapsed into three categories (“strongly agree/agree”, “uncertain”, and “disagree/strongly disagree”). Differences in mean scores on subscales (i.e., total score on items assessing attitudes) between participant groups (i.e., those with/without past training) were examined using independent Student’s *t*-tests.

## 3. Results

The majority of the 281 mental health clinicians were female (72.6%), approximately 40 years of age, with a mean practice experience of 12.1 years. The most common profession was nursing (reported by just under 70% of respondents), followed by medical staff, support workers, social workers, psychologists, and occupational therapists. The majority of participants worked at public or private mental health services for adults, with the remainder working in Mental Health Community Support Services (MHCSS)/Psychiatric Disability Support Services (PDRSS) or alcohol and other drug (AOD) services (Table 1).

### 3.1. Gambling Problems as a Percentage of Clinician Caseloads

On average, clinicians estimated that 11.01% of their caseloads included patients with gambling problems (SD *=* 11.38%, range 0%–65%). Almost all participants (82.6%) reported that at least one patient in their caseload was experiencing gambling problems. Clinicians reported a higher percentage of gambling problems in their caseloads (M = 13.72%, SD = 13.64%) compared to female clinicians (M = 9.99%, SD = 10.26%), *t*(276) = 2.46, *p* = 0.009, 95% CI [0.71, 6.73].

### 3.2. Previous Training in Problem Gambling

Of the full sample of 281 clinicians, only 35 (12.5%) reported ever engaging in training in screening and responding to PG. There was a significant difference in rates of previous training across the professions listed in Table 1 (χ^2^ = 15.58, *p* = 0.016), with a higher rate of training reported by psychologists (41.2%) compared to nurses (11.5%), medics (13.3%), social workers (12.9%), support workers (8.9%), and occupational therapists (OTs; 11.8%). No participants in the “other” profession category reported received training in PG.

### 3.3. Gambling and Mental Illness Knowledge

Clinicians’ knowledge of the bidirectional relationship between PG and mental illness was assessed by their response to four items (Table 2). The majority of clinicians demonstrated a good understanding of the comorbidity of PG and mental illness and the impact of PG on the severity of a patient’s mental illness. However, less than half of the sample agreed or strongly agreed that they understood what causes and/or maintains PG issues, while the majority was not aware of screening and assessment tools available for the detection of PG. Those who had previously received training in PG were significantly more likely to agree/strongly agree that PG and mental illness commonly co-occur (χ^2^ (2) = 7.173, *p* = 0.028) and that they understood what causes and/or maintains PG issues (χ^2^ (2) = 14.681, *p* = 0.001).

### 3.4. Attitudes Towards Responding to Gambling Issues

Clinicians’ responses to items assessing attitudes towards responding to gambling issues are presented in Table 3. The mean total score was 41.4 (SD = 5.03), with individual scores ranging from 20 to 53 (with a maximum possible score of 55; lower scores indicate a reluctance to deal with gambling issues). This finding suggests that, overall, clinicians indicated being willing to address gambling issues with their patients when these arose. Clinicians who had received training had significantly more positive attitudes towards responding to gambling issues (M = 43.29, SD = 4.38) compared to those who had not received training (M = 41.16, SD = 5.04), *t*(275) = 2.266, *p* = 0.019. Surprisingly 20% indicated that they do not consider gambling disorder as a mental health disorder. Although 75% agreed with the statement “a brief problem gambling screen would be a useful part of my routine clinical practice”, a substantial proportion indicated that there were more important issues and insufficient time to screen for PG.

### 3.5. Comorbidity

**Figure 1:**
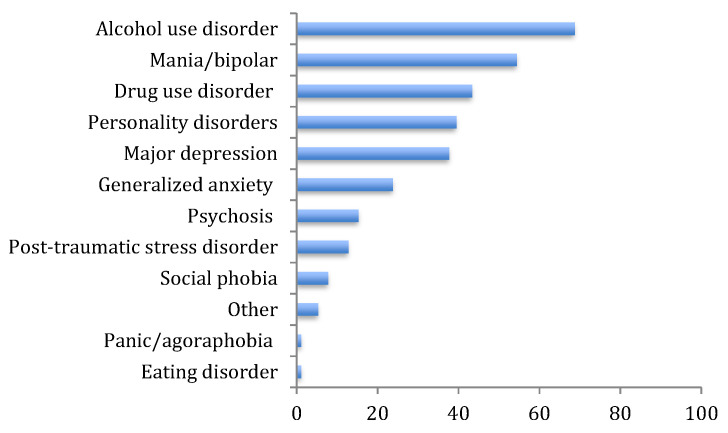
Mental health disorders where gambling problems most frequently occur according to clinicians.

Clinicians were asked to identify the most common mental health disorders seen in their practice where comorbid PG is observed. Clinicians reported that, in their experience, PG most commonly occurs with alcohol use disorder (68.7%), followed by mania/bipolar disorder (54.4%), then drug use disorder (43.4%), personality disorders (39.5%), and major depression (37.7%) (Figure 1).

### 3.6. Screening and Assessment

The majority of clinicians (76.9%) reported screening for PG; however, only 16.0% reported screening “often” or “almost always” and over 60.9% reported doing it only “sometimes” or “rarely”, whilst 22.4% reported “never” screening. Clinicians with previous training were more likely to screen for PG (χ^2^ = 9.56, *p* = 0.049) compared to clinicians who had not received training.

The majority of clinicians (85.7%) who reported screening their patients for gambling problems did so informally during an appointment or interview, with 6.7% reporting the use of set questions in their service’s intake assessment and only 2% reporting the use of a standardized or formal self-report PG screening tool.

Most clinicians reported feeling comfortable (39.1% “very comfortable” and 43.8% “somewhat comfortable”) asking patients about their gambling behavior, with 13.2% feeling “somewhat uncomfortable” and 3.9% feeling “very uncomfortable”. Fewer were confident in detecting/screening for gambling problems (4.6% “very confident”, 29.5% “moderately confident”, and 39.1% “somewhat confident”), with just over a quarter (25.6%) reporting they were not confident. The proportion of clinicians who were very/somewhat comfortable asking patients about their gambling behavior did not differ (81.3% vs 83.1%) between those who had received training and those who had not (χ^2^ (1) = 0.803, *p* = 0.477). The proportion of clinicians very/moderately confident in detecting/screening for PG was significantly greater (56.3% vs. 34.0%) among those who had received training (χ^2^ (1) = 5.78, *p* = 0.014).

### 3.7. Actions Taken Following Identification

Clinicians reported a range of actions taken after identifying a patient with gambling problems (multiple actions could be selected if applicable). A majority (69.0%) reported referring the patient to an external treatment provider, while just under half (46.3%) addressed the financial or social consequences of gambling. Approximately one-third conducted further assessment (38.8%) or psychological treatment (23.5%), while a minority (2.8%) provided pharmacotherapy. Interestingly, a considerable minority (13.5%) reported that they had never identified a patient with gambling problems.

### 3.8. Referral

Most clinicians (90.0%) agreed/strongly agreed that it was important to refer patients who were experiencing gambling problems to specialist agencies for further treatment. The majority indicated that they had referred patients with a gambling problem to other services for help, though this occurred infrequently (41.3% referred “rarely”, while 29.5% referred “sometimes”). Only 33 (11.7%) referred “almost always” or “often”, while 45 (16.0%) reported never referring patients to other services for help with their gambling (1.4% had missing data). The most common referrals made were to gambling helplines (142; 50.5%) and face to face gambling help services (139; 49.5%), followed by financial counselling (98; 34.9%), Gamblers Help Online (95; 33.8%), Gamblers Anonymous/peer support (64; 22.8%), private psychologists/psychiatrists (38; 13.5%), and hospital-based specialist gambling service (30; 10.7%). Referral to private addiction/gambling therapists (20; 7.1%) and referral to “other” services (17; 6.0%) were the least common referral pathways for patients experiencing PG (note that participants could select multiple responses).

### 3.9. Treatment

A majority (77.5%) of clinicians reported that they did provide treatment for gambling problems where needed, with 6.0% treating patients “often”, 29.9% treating patients “sometimes”, and 41.6% treating patients “rarely” (20.6% never treated patients, while 1.8% had missing data). Treatment was mostly provided in the form of counselling (35.2%) or assessment only (39.5%). Less than 20% reported offering peer support, medication, financial counselling, or financial aid/relief. Almost half of participants (46.6%) were “not confident” in treating gambling problems, 29.5% were only “somewhat confident”, 18.5% were “moderately confident”, and 2.8% were “very confident”. Participants indicated having limited understanding of the gambling service system and programs and which treatments are most effective (Table 4). Finally, most clinicians (83.3%) agreed or strongly agreed that mental health clinicians can effectively work together to support patients.

### 3.10. Differences in Clinician Response by Profession

The sample was too small to permit statistical comparisons across the eight profession groups; however, when split by medical staff (i.e., doctors and nurses; 49.9%) versus nonmedical staff (i.e., all other professions; 49%), we found no differences in any of the reported responses, with the exception of nonmedical professionals reporting significantly more positive attitudes towards responding to gambling issues (*t*(275) = −2.299, *p* = 0.02). Similarly, there were too few clinicians (*n* = 10) from private mental health services for any statistically meaningful comparisons in response by mental health service type.

## 4. Discussion

The results of the survey indicated that mental health clinicians recognize the importance of responding to gambling issues. However, while they displayed high levels of knowledge, their confidence in responding to PG was generally low, as was the frequency with which they screen for PG. This is of concern given previous studies have concluded that mental health clinicians have a significant role in the identification and management of PG [20]. Indeed, the majority of the current sample reported that 10%–25% of their caseload was affected by gambling. Shame, denial, and stigma are common reasons why people with gambling problems are reluctant to seek treatment [29], with an estimated five-year latent period between the development of a problem and professional help-seeking [29,31,39,40,41,42]. As mental health clinicians are already highly skilled in discussing sensitive issues in stigmatized populations (e.g., suicidal thoughts, family violence, etc.), they are ideally placed to explore their patients’ gambling behavior. It was therefore not surprising that clinicians expressed confidence in asking a patient about their gambling behavior; however, it is critical that this happens routinely and that confidence in managing/treating gambling problems is increased to ensure an efficient and effective response to patients with comorbid mental health and gambling issues.

It is likely that the lack of confidence in managing PG reflects low rates of previous training in PG. This is not surprising given the assessment and management of PG is not routinely taught within undergraduate or postgraduate healthcare training programs, nor is it a competency requirement to work within mental health settings. Inadequate access to training and education resources has been identified as a key factor underlying low confidence in identifying and managing PG [43] and is consistent with previous research investigating screening by healthcare workers and their training needs, knowledge, and skills [44]. With only 16% “often” or “almost always” undertaking screening, it is likely that many gambling issues go undetected in mental health services, resulting in missed opportunities for early support and treatment. Gambling screening is particularly pertinent in a population where lower disposable income and financial stress are commonplace [36] and where gambling losses are likely to have an adverse impact. Research suggests people with gambling problems are 15 times more likely to attempt suicide, with the greatest increase in risk (19 times) among men aged between 29 and 40 [45]. As such, early identification and support must be offered to individuals where mood instability and poor impulse control could leave them more susceptible to personally harmful behaviors.

Addressing the training gap is critical because research suggests that service provider training in other areas (e.g., substance use and mental health) is a determinant of whether or not clinicians screen for such issues [46]. At a minimum, PG training should discuss the relationship between mental health and gambling as well as guidelines and approaches related to assessment and management [45,47]. However, consistent with competency training for other health conditions, PG training should support clinicians to develop core clinical skills with opportunities to practice and implement what has been taught under accredited supervision. In the current study, the most common method for the identification of gambling problems was via informal conversations, discussions, or questions in sessions or intake assessments. Only a minority reported using questions included in their service’s intake assessment or using standardized or formal self-reported PG screening tools, such as the PGSI. However, clinicians could see the value in screening for PG and indicated that standardized screening tools should be used even if patients do not mention PG themselves. We have shown that brief two-item screening tools (such as the Lie/Bet and two-item Brief PG Screen) adequately detected PG in mental health patients in these Australian services, with diagnostic efficiency exceeding 0.90 [48]. There is also evidence from a recent meta-analysis based on international evidence that the two-item Lie/Bet and three-item Brief Biosocial Gambling Screen display satisfactory diagnostic accuracy when used in nongambling clinical settings [49]. Given the indicated importance of other screening and assessment priorities with a mental health patient population, having reliable and brief screen tools is imperative. As such, we recommend embedding one of these brief screening tools to intake and assessment forms in mental health services where data on alcohol, tobacco, and illicit drug use is routinely collected. It may benefit clinicians to preface any PG screening questions with a statement around the routine nature of enquiry concerning addiction/lifestyle behavior as well as the safety, confidentiality, and potential advantages of any disclosures in terms of assisting with treatment or support.

Barriers and facilitators to screening (e.g., time) are likely to exist at a system or agency level as well as clinician and patient levels [50]. Funding for the management of PG in mental health services can act as a facilitator in terms of addressing competing priorities and raising the importance or profile of PG among mental health patients. For example, in recent years, there has been considerable investment in building mental health clinician capacity in responding to alcohol and drug issues across Australia, as evidenced by the development and aims of the National Comorbidity Guidelines, in addition to state-specific strategies, such as the Victorian Dual Diagnosis Initiative [28] and the Victorian Government’s “no wrong-door” investment [36]. Such initiatives have seen an increase in mental health workforce capacity to deal with substance use issues as well as greater utilization of routine mental health screening across the alcohol and other drug sector.

With respect to actions taken when a patient with a gambling problem is identified, only 40% of clinicians reported referring patients to external services. While low referral rates may reflect the absence of routine screening, it may also reflect a lack of understanding of what services are available and what they can offer, particularly in terms of managing complex comorbid patients. This could potentially be improved by addressing barriers to service integration, including those related to funding, as well as facilitating integration at an organizational level by developing interagency relationships, shared organizational purpose, values, and priorities, and the colocation of services. Other strategies to facilitate integrated working include those at the level of service delivery (e.g., staff training, information sharing, referral, and the development of staff interprofessional networks) as well as clinical strategies (e.g., screening, joint care planning, and supervision) [51,52].

While most clinicians reported providing treatment for patients identified as problem gamblers, few (6%) reported doing so often. In addition, low confidence in treatment provision and knowledge of effective treatments for PG was evident, which is consistent with the findings of Achab and colleagues (2014). When PG is identified, clinicians need to be able to offer or refer to evidence-based brief interventions. These could be provided by mental health clinicians or alternatively via online or app-based self-directed programs [53], which is an emerging area of service provision for PG.

Whilst the findings inform implications for improved practice, it is important to acknowledge a number of limitations. These include the potential for self-selection bias among participating clinicians who may have greater interest or experience with PG; it should, however, be noted that over 70% of eligible clinicians were sampled, which minimizes the likelihood of sampling bias. Clinicians also self-reported their attitudes and behaviors rather than being observed in practice, and social desirability effects may therefore have influenced their responses. Nonetheless, a large number of clinicians were recruited from a wide range of mental health services and multiple different service sites across Victoria. The findings presented reflect data collected five years ago, and further research is needed to determine whether clinician attitudes or behaviors have subsequently changed or if these vary by professional background. A key finding was that clinicians who had received training in PG reported greater confidence in responding; however, only a handful of respondents provided details on the nature of this training, and it is likely to have varied greatly in its scope and duration. Despite such limitations, this is the first study to examine the way in which mental health clinicians respond to problem gambling issues and provides a valuable snapshot of not only training needs but also opportunities for enhanced integrated working practices with gambling services.

## 5. Conclusions

In summary, in light of the elevated rates of gambling harm experienced among people attending mental health services [54], these findings suggest mental health clinicians may need to offer strategies and interventions tailored to mental health populations that reduce gambling-related harm as well as resources, support, and referral options. At a minimum, clinicians should routinely ask patients about their gambling behavior, and treatment services should consider embedding a brief PG screening tool into intake assessment documentation. Beyond screening and referral practices, there is also a need for greater focus on training and education initiatives concerning PG management across the mental health sector as well as integrated working practices with gambling services [55].

## Figures and Tables

**Table 1:** Clinician demographics.

Total Sample (*n* = 281)	
Age (years), mean (range)	40.10 (20–67 years)
Male (*n*, %)	77 (27.4)
Profession (*n*, %)	
Medical	62 (22.1%)
Nurse	78 (27.8%)
Social worker	31 (11.0%)
Psychologist	17 (6.0%)
Occupational therapist	17 (6.0%)
Support worker	56 (19.9%)
Other	17 (6.0%)
Missing	3 (1.1%)
Type of service (*n*, %)	
Public mental health service (adult)	203 (72.3%)
Private mental health service	10 (3.6%)
Primary healthcare	23 (8.2%)
MHCSS/PDRSS	45 (16.0%)
Practice duration (years), mean (range)	12.1 (<1 year–40 years)

**Table 2:** Self-reported knowledge of gambling (*n* = 281).

	Strongly Agree/Agree	Uncertain	Disagree/Strongly Disagree	Missing
Problem gambling and mental illness commonly occur together	178 (63.3%)	83 (29.5%)	20 (7.1%)	0
Problem gambling can worsen a patient’s mental illness	265 (94.3%)	14 (5.0%)	1 (0.4%)	1 (0.3%)
I understand what causes and/or maintains problem gambling issues	125 (44.4%)	115(38.9%)	40 (14.2%)	1 (0.4%)
I am aware of screening and assessment tools available for detection of problem gambling	32 (11.4%)	64 (22.8%)	184 (65.4%)	1 (0.4%)

**Table 3:** Attitudes towards responding to gambling issues.

	Strongly Agree/Agree	Uncertain	Strongly Disagree /Disagree	Missing
There is no point conducting gambling screening as my service does not treat problem gamblers	13 (4.7%)	29 (10.3%)	237 (84.3%)	2 (0.7%)
Gambling disorder is not really a mental health disorder	17 (6.1%)	44 (15.7%)	219 (78.0%)	1 (0.4%)
Detecting problem gambling does not require a formal screen; it can just be addressed if a patient mentions it	40 (14.2%)	56 (19.9%)	183 (65.1%)	2 (0.7%)
Use of standardized screening tools is only necessary if a patient mentions gambling	34 (12.1%)	70 (24.9%)	173 (61.6%)	4 (1.4%)
People accessing mental health treatment do not want to be screened for gambling problems	27 (9.6%)	101 (35.9%)	151 (53.7%)	2 (0.7%)
There are too many more important issues to screen for problem gambling	30 (10.7%)	41 (14.6%)	207 (70.7%)	3 (1.1%)
Problem gambling does not co-occur with mental health problems often enough to bother screening	8 (2.8%)	50 (17.8%)	220 (78.3%)	3 (1.1%)
There is not enough time to conduct problem gambling screening or assessment in my workplace	65 (20.9%)	75 (24.1%)	167 (53.7%)	4 (1.3%)
Screening/assessment and referral for problem gambling is not part of my job	20 (7.1%)	36 (12.8%)	222 (79.0%)	3 (1.1%)
It is important to identify gambling problems among mental health patients	253 (90%)	13 (4.6%)	12 (4.9%)	1 (0.4%)
A brief problem gambling screen would be a useful part of my routine clinical practice	111 (75.1%)	43 (15.3%)	26 (9.2%)	1 (0.4%)

**Table 4:** Current treatment practices and knowledge about treatment options.

Sample (*n* = 281)	Strongly Agree/Agree	Uncertain	Disagree/Strongly Disagree	Missing
I have a good understanding about the Gambler’s Help service system and the programs available	65 (23.2%)	65 (23.1%)	150 (53.3%)	1 (0.4%)
I understand the types of treatments that have proven helpful for PG	62 (22.0%)	79 (28.1%)	129 (49.5%)	1 (0.4%)
Mental health and PG clinicians can effectively work together to support patients	134 (83.3%)	35 (12.5%)	10 (3.5%)	2 (0.7%)
